# The effect of pegbovigrastim administration on the nonspecific immunity of calves

**DOI:** 10.1111/jvim.16939

**Published:** 2023-12-05

**Authors:** Katarzyna Dudek, Ewelina Szacawa, Dariusz Bednarek

**Affiliations:** ^1^ Department of Cattle and Sheep Diseases National Veterinary Research Institute Pulawy Poland

**Keywords:** cytokines, leukocyte surface markers, leukocytes, myeloperoxidase, oxygen burst, phagocytic activity

## Abstract

**Background:**

Prevention of diseases in the early rearing of calves is important, particularly because disease occurrence most often requires antimicrobial administration but reduction of their use in animals is a priority. Pegbovigrastim is known for its use as an immunoregulator in cows and heifers, but the effect of its administration on calves has not been fully investigated.

**Objectives:**

Investigate whether administration of pegbovigrastim effectively stimulates nonspecific immunity in healthy calves.

**Animals:**

Eleven clinically healthy 5‐week‐old calves.

**Methods:**

Prospective observational study. Calves were randomly allocated to an experimental or control groups to receive pegbovigrastim or the same volume of phosphate‐buffered saline twice over a 7‐day period. To evaluate nonspecific immunity, the numbers of total leukocytes and cells in the appropriate cell fractions were determined. Cytometric analyses were carried out to identify cells expressing CD11b and to evaluate the phagocytic and oxidative burst activities of granulocytes and monocytes. Myeloperoxidase (MPO) and selected cytokines were assayed using ELISA.

**Results:**

Pegbovigrastim significantly increased the number of total leukocytes and of cells in all of the examined subsets (*P* < .05). The phagocytic activity of leukocytes expressed as mean fluorescence intensity was significantly potentiated after pegbovigrastim administration (*P* < .05). The cytokine response was modulated by pegbovigrastim administration toward anti‐inflammatory activity.

**Conclusions and Clinical Importance:**

Pegbovigrastim effectively stimulated nonspecific immunity in clinically healthy calves, which in the long term could make the prevention of diseases during early rearing possible by strengthening the immune defense mechanisms of the host.

**Abbreviations**
IL‐10: interleukin‐10
IL‐1RA: interleukin‐1 receptor antagonist
IL‐1β: interleukin‐1β
IL‐6: interleukin‐6
MFI: mean fluorescence intensity
MPO: myeloperoxidase
TNF‐α: tumor necrosis factor‐α

## 1 INTRODUCTION

Colony‐stimulating factors (CSFs) are agroup of cytokines responsible for hematopoiesis, the examples being different depending on the type of target progenitor cells. Among CSFs, granulocyte colony‐stimulating factor (G‐CSF) is responsible for the regulation of granulopoiesis, which it does by stimulating proliferation and differentiation of progenitor cells in the bone marrow.^1^ It is known that G‐CSF affects some human neutrophil functions in vitro, including antibacterial activity.^2^ A previous study showed that recombinant bovine G‐CSF (rbG‐CSF) modulated the expression of some surface antigens on activated bovine neutrophils in vitro.^3^ Pegbovigrastim is a pegylated form of bovine G‐CSF (bG‐CSF) used primarily in preventing or treating mastitis in dairy cows.^4^, ^5^, ^6^, ^7^ Numerous studies in adult cattle have shown a stimulating effect of pegbovigrastim on total and differential leukocyte counts.^5^, ^8^, ^9^, ^10^, ^11^, ^12^ However, little is known about the effect of pegbovigrastim on calves, because only a few studies have been conducted in this area.^13^, ^14^, ^15^ In 1 such study, pegbovigrastim administered to newborn calves caused significant increases in the numbers of circulating leukocytes and some white blood cell subtypes. General stimulation of the leukocyte response also was observed in calves aged between 30 and 60 days under conditions of induced endotoxemia.^15^ Our previous preliminary study showed a general stimulating effect of pegbovigrastim administration to calves on the numbers of granulocytes and monocytes and their phagocytic and oxidative burst activities despite ongoing *Mycoplasma bovis* infection.^14^ In the studies cited above, the assessment of these parameters in healthy calves was only short‐term (until infection with *M. bovis*) or involved only 1 injection of pegbovigrastim.^14^, ^15^ Information still is limited on the changes in or relationships among these parameters in clinically healthy calves in response to multiple injections of pegbovigrastim designed to mimic the administration protocol used in adult periparturient cows. Knowledge of these changes is important to understand how pegbovigrastim's effect interacts with differences in the development of the immune system between pre‐weaned calves and periparturient dairy cattle. Therefore, our objective was to investigate whether pegbovigrastim given twice effectively stimulates the main parameters of nonspecific immunity, namely numbers of total leukocytes and of cells in the appropriate fractions, percentage of cells expressing CD11b, phagocytic and oxidative burst activities of granulocytes and monocytes and concentrations of myeloperoxidase (MPO) and selected cytokines in healthy calves, most of which have not yet been determined under these conditions.

## 2 MATERIALS AND METHODS

The experimental procedures were carried out in accordance with the requirements of the Local Ethics Committee on Animal Experimentation of the University of Life Sciences in Lublin, Poland (Resolution No. 33/2018 passed on 12 February 2018), which also meet the European Union (EU) standards.

### 2.1 Animals and pegbovigrastim administration

The study was carried out on 11 clinically healthy female calves of the Holstein‐Friesian breed at a mean age of 5 weeks. After an adaptation period that lasted 4 weeks, the calves were randomly allocated to 2 groups: experimental (E, n = 6) and control (C, n = 5). They were housed in the vivarium at the National Veterinary Research Institute in Pulawy, Poland in 4 pens (2 pens holding 3 animals each for the E group and 2 pens holding 3 animals in 1 case and 2 animals in the other for the C group) in a shared air space. The animals received milk replacer twice a day and hay and water ad libitum. The E group was injected SC with pegbovigrastim (Imrestor, Eli Lilly and Company Limited, Elanco Animal Health) at a single dose of 40 μg/kg body weight (bw) on day 0 and again on day 7. The C group received sterile phosphate‐buffered saline, pH 7.2, instead. Blood samples were collected on day 0 (just before the first administration of pegbovigrastim), on days 2 and 5 after this administration, on day 7 (just before the second administration of pegbovigrastim), and then on days 2, 5, 7, and 14 after this administration (ie, on days 9, 12, 14 and 21 after the first administration of pegbovigrastim). Blood samples were collected from a jugular vein of each animal into 1 of 3 types of tube, depending on the particular analysis intended. These were a tube coated with EDTA for hematological analysis and CD11b antigen determination, a tube with heparin for the determination of phagocytic and oxidative burst activities of peripheral blood granulocytes and monocytes, and a tube coated with clot activator or gel for serum separation intended for use in determinations of MPO, interleukin‐1 beta (IL‐1β), tumor necrosis factor alpha (TNF‐α), interleukin‐6 (IL‐6), interleukin‐1 receptor antagonist (IL‐1RA), and interleukin‐10 (IL‐10). To obtain sera, the tubes were centrifuged at 1500*ɡ* for 10 minutes at 18°C to 25°C. Blood samples intended for hematological and cytometric analyses were used immediately after the collection, whereas serum samples were stored at −20°C ± 5°C until MPO and cytokine concentrations were analyzed.

### 2.2 Total and differential leukocyte count

The numbers of total leukocytes and of cells by subpopulations such as granulocytes, lymphocytes, and monocytes were counted using an automatic veterinary blood analyzer (Exigo, Boule Medical AB, Sweden).

### 2.3 CD11b antigen

The CD11b cell surface antigen on granulocytes and monocytes was determined in whole blood using a monoclonal antibody (mouse anti‐bovine CD11b : FITC, integrin alpha m chain, MAC‐1, clone CC126, isotype IgG2b, Bio‐Rad Laboratories Inc) according to the procedure for phenotyping of bovine peripheral blood T cells as described previously.^16^ Analysis was performed using a flow cytometer (Epics XL, Beckman Coulter, Florida).

### 2.4 Phagocytic and oxidative burst activities of granulocytes and monocytes

The phagocytic and oxidative burst activities of peripheral blood granulocytes and monocytes were quantified using commercially available kits as described previously^17^, ^18^ and analyzed using the Epics XL flow cytometer (Beckman Coulter, Florida).

### 2.5 Myeloperoxidase and cytokine concentrations

The concentrations of MPO and selected cytokines such as IL‐1β, TNF‐α, IL‐6, IL‐1RA, and IL‐10 were measured using separate commercially available ELISA kits (Cloud‐Clone Corp.) according to the manufacturer's instructions. The optical densities in the microwells for each parameter were determined at 450 nm and read by an automated plate reader (Elx800 Microplate Reader, BioTek Instruments, Inc, USA) using the KC Junior program (BioTek Instruments, Inc, USA).

### 2.6 Statistical analyses

Statistical analysis was performed using Statistica Desktop 13 (StatSoft Polska Sp. z o.o., Poland). Results are presented as arithmetic mean or mean percentage and SD. Data were evaluated for normal distribution using the Shapiro‐Wilk test and for homogeneity of variances by Levene's test. The differences between the mean values recorded in the experimental group and those found in the control groups at the same time point were analyzed using 1‐way ANOVA. A Tukey's post hoc test was performed to compare several groups. For a few results that were not normally distributed, the nonparametric Kruskal‐Wallis ANOVA and median tests were used. A *P*‐value <.05 was considered significant in all cases.

## 3 RESULTS

### 3.1 Total and differential leukocyte count

**FIGURE 1:**
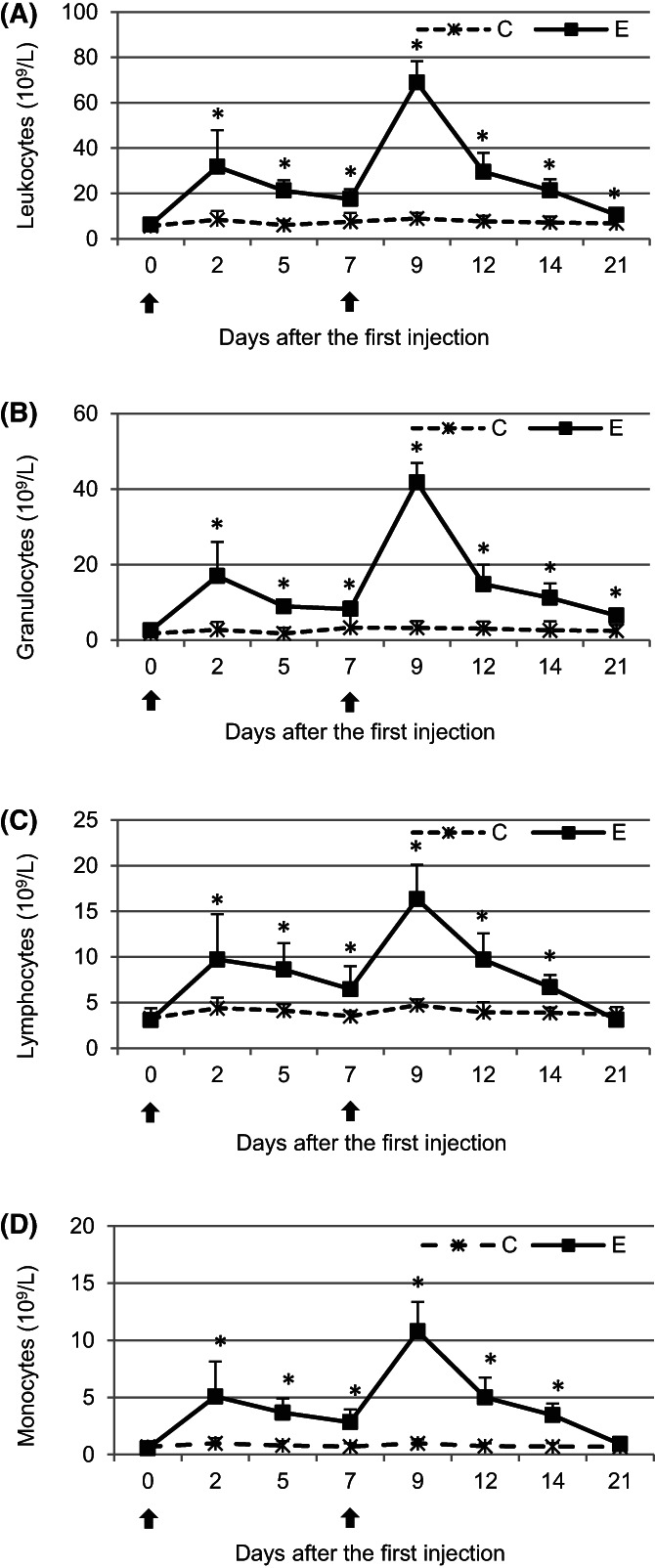
The mean (±SD) number of total leukocytes (A) and numbers of cells in granulocyte (B), lymphocyte (C), and monocyte (D) subpopulations in the peripheral blood of calves after administration of pegbovigrastim. E—experimental group; C—control group; arrow—pegbovigrastim injection; * significant difference (*P* < .05) between the E and C groups.

On days 2, 5, and 7 after the first administration of pegbovibrastim significant increases (*P* < .05) in total leukocyte, granulocyte, lymphocyte, and, monocyte counts were observed in E group when compared with those in C group. These increases were more pronounced after the second administration of pegbovigrastim and the counts remained significantly higher (*P* < .05) than those in the C group until the end of the study on day 21 for total leukocyte and granulocyte counts or until day 14 for the lymphocyte and monocyte counts (Figure 1A‐D).

### 3.2 CD11b antigen

**FIGURE 2:**
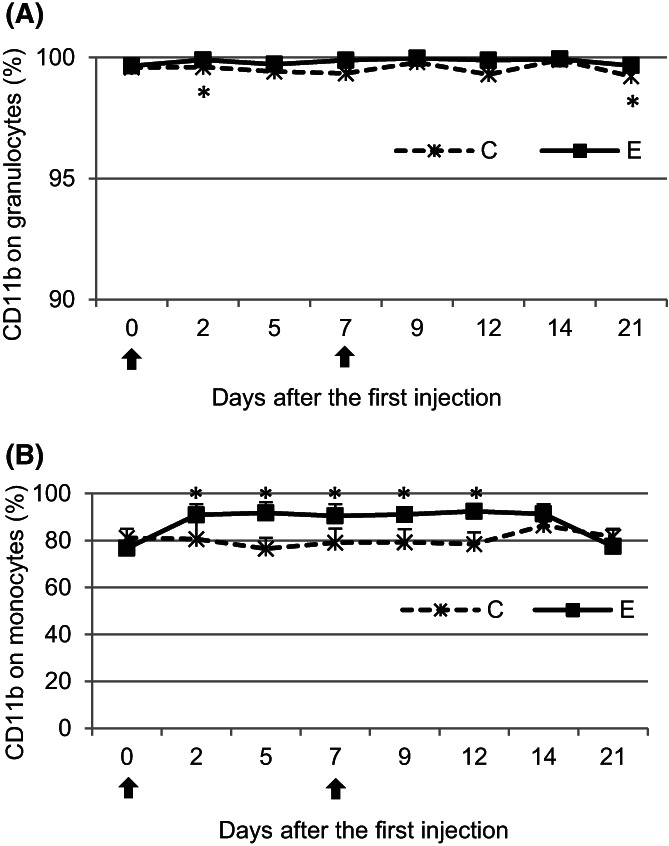
The mean (±SD) percentage of CD11b antigen on granulocytes (A) and monocytes (B) in the peripheral blood of calves after administration of pegbovigrastim. E—experimental group; C—control group; arrow—pegbovigrastim injection; * significant difference (*P* < .05) between the E and C groups.

The percentages of CD11b‐bearing granulocytes and monocytes in the E group were higher than those in the C group throughout the study, with the exceptions of days 0 and 21 for monocytes. Significant (*P* < .05) differences in the percentages between the examined groups were observed on days 2 and 21 for granulocytes and on days 2, 5, 7, 9, and 12 for monocytes (Figure 2).

### 3.3 Phagocytic activity of granulocytes and monocytes

**FIGURE 3:**
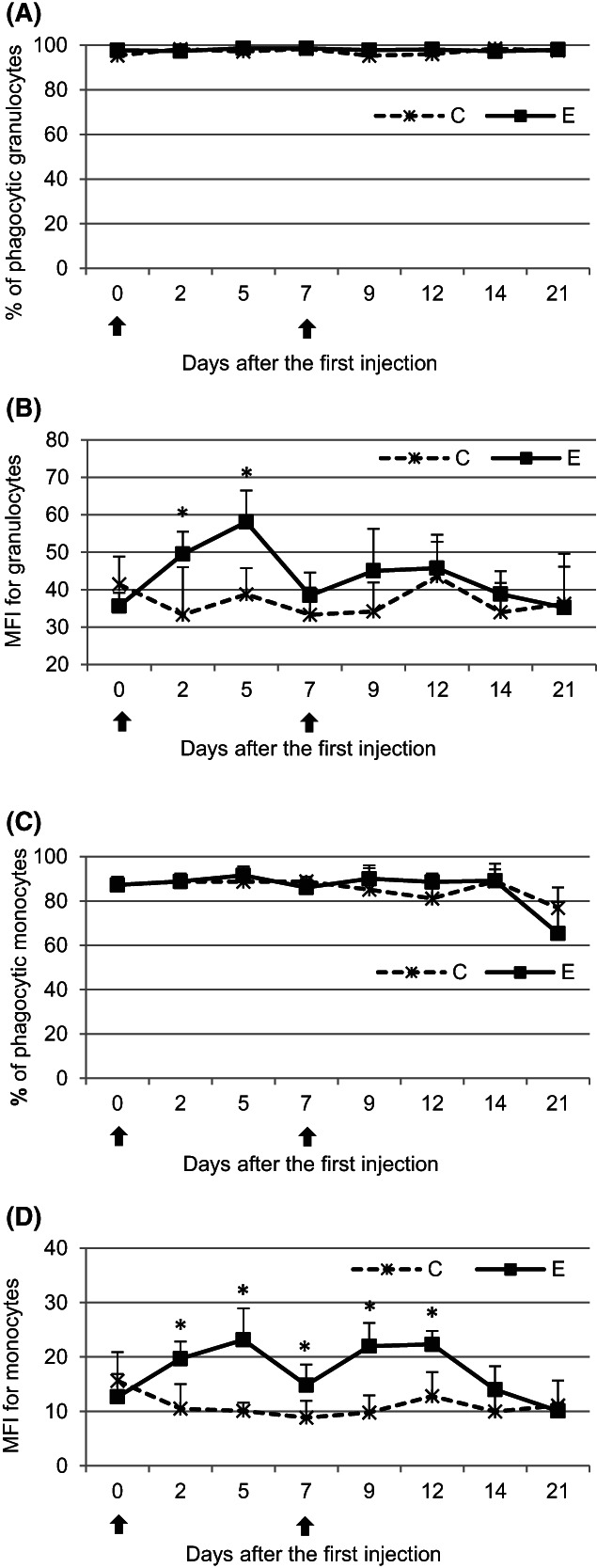
The mean (±SD) phagocytic activity of granulocytes (A,B) and monocytes (C,D) expressed as the mean percentage of phagocytic cells and mean fluorescence intensity (MFI) in the peripheral blood of calves after administration of pegbovigrastim. E—experimental group; C—control group; arrow—pegbovigrastim injection; * significant difference (*P* < .05) between the E and C groups.

After the first administration of pegbovigrastim, no significant (*P* < .05) differences in the percentages of phagocytic granulocytes and monocytes were observed when comparing the E group to the C group. Also, none were observed after the second administration (Figure 3A,C). Nevertheless, on days 2 and 5 after the first administration of pegbovigrastim, the mean fluorescence intensity (MFI) for granulocytes was significantly (*P* < .05) higher in the E group than in the C group. At the remaining time points, no significant differences (*P* < .05) between the groups were observed (Figure 3B). The MFI for monocytes significantly (*P* < .05) increased in the E group on days 2, 5, and 7 after the first injection of pegbovigrastim when compared with the C group. The intensity continued to be higher after the second administration and remained significantly (*P* < .05) so in comparison to that of the C group until day 12 of the study (Figure 3D).

### 3.4 Oxidative burst activity of granulocytes and monocytes

**FIGURE 4:**
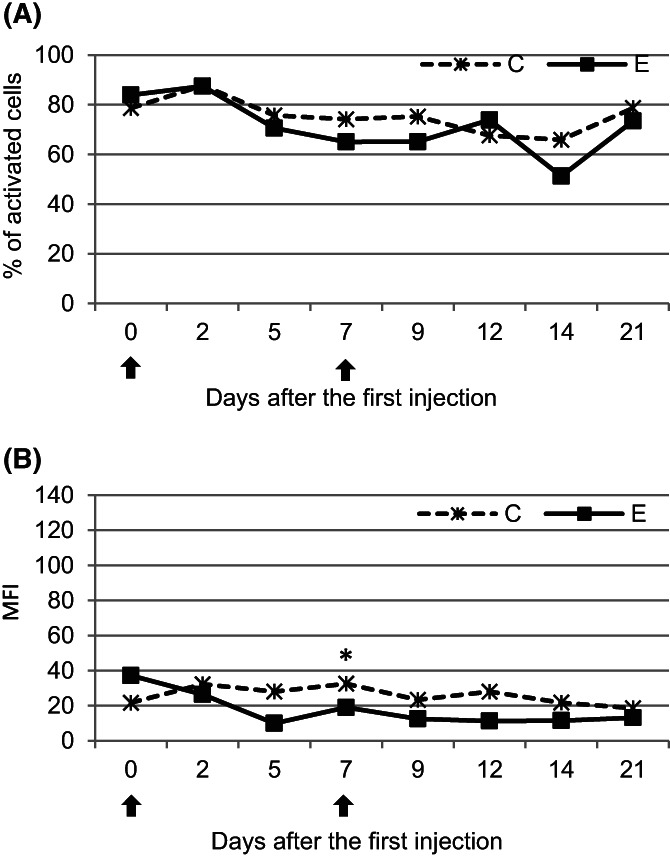
The mean (±SD) oxidative burst activity of leukocytes expressed as the mean percentage of cells and mean fluorescence intensity (MFI) in the peripheral blood of calves after administration of pegbovigrastim. E—experimental group; C—control group; arrow—pegbovigrastim injection; * significant difference (*P* < .05) between the E and C groups.

No significant (*P* < .05) differences between the examined groups were observed throughout the study in the percentages of activated cells or MFI values, with the exception of MFI on day 7 when significantly (*P* < .05) lower values were found in the E group (Figure 4A,B).

### 3.5 Myeloperoxidase concentration

**FIGURE 5:**
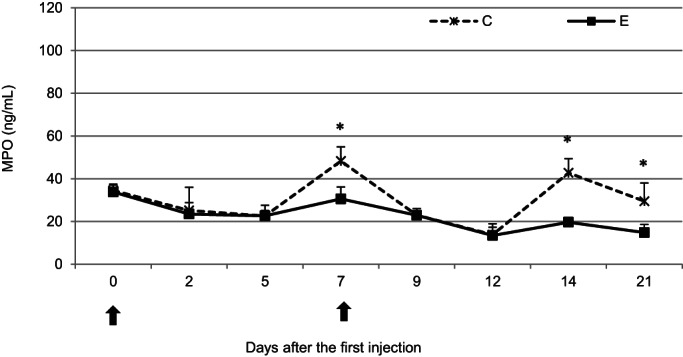
The mean (±SD) concentration of myeloperoxidase (MPO) in the serum of calves after administration of pegbovigrastim. E—experimental group; C—control group; arrow—pegbovigrastim injection; * significant difference (*P* < .05) between the E and C groups.

Neither administration of pegbovigrastim caused detectable changes in the MPO concentration despite this parameter undergoing marked changes in the untreated C group, in which the concentration significantly (*P* < .05) increased on days 7, 14, and 21 of the study. At the remaining time points, the MPO concentrations were comparable between the groups (Figure 5).

### 3.6 Cytokine concentrations

**FIGURE 6:**
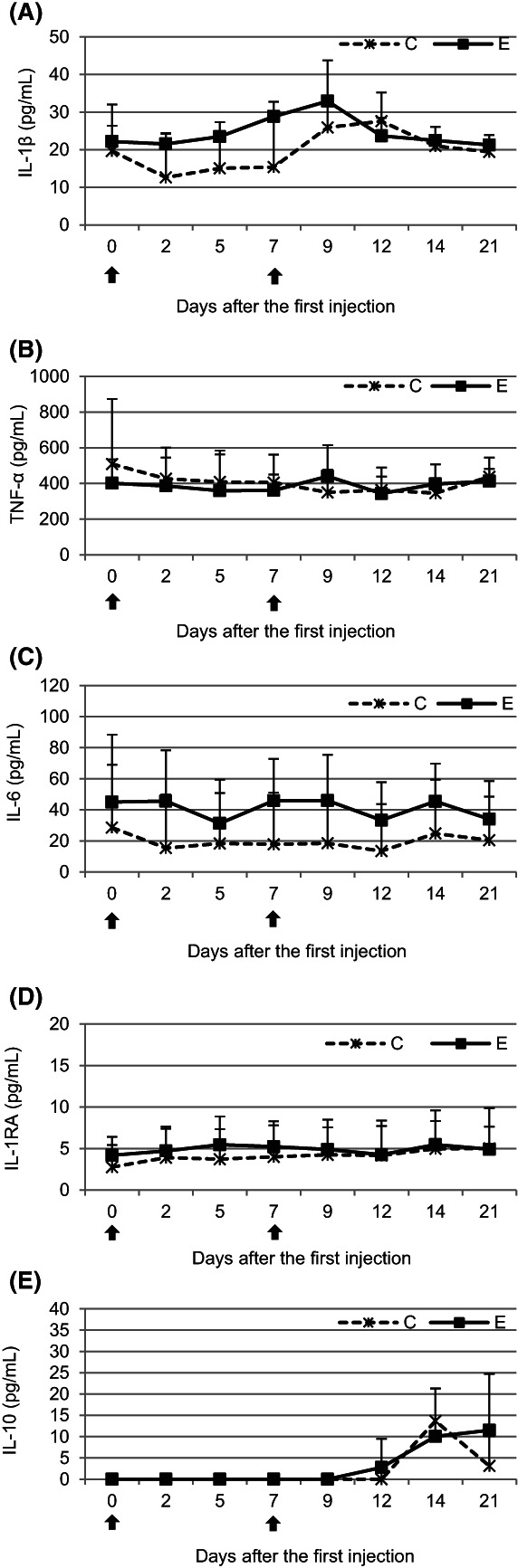
The mean (±SD) concentration of IL‐1β (A), TNF‐α (B), IL‐6 (C), IL‐1RA (D), and IL‐10 (E) in the serum of calves after administration of pegbovigrastim. E—experimental group; C—control group; arrow—pegbovigrastim injection.

The first administration of pegbovigrastim caused a detectable increase in the IL‐1β concentration until day 7 when comparing the E group to the C group. After the second administration, an increase in this parameter was observed on day 9 of the study. At the remaining time points, the IL‐1β concentrations were comparable between groups. No significant (*P* < .05) differences between the examined groups were observed throughout the study (Figure 6A).

An increase in the IL‐6 concentration in the E group was observed throughout the study when compared with the C group, but this increase did not represent a significant (*P* < .05) difference (Figure 6C).

The concentrations of TNF‐α, IL‐1RA, and IL‐10 were comparable between the groups throughout the study, with the exception of IL‐10 on day 21 when an increased concentration was observed in the E group (Figure 6B,D,E).

## 4 DISCUSSION

We evaluated the effect of pegbovigrastim administration in clinically healthy calves on the expression of CD11b, the concentration of MPO, and selected cytokines.

Phagocytes are the major cells involved in nonspecific immunity, which is the first line of host defense against infection, activated shortly after exposure to a pathogen. Being phagocytes, neutrophils are involved primarily in phagocytosis and oxidative burst, key mechanisms of the host antimicrobial defense.^19^

Previous studies have shown a stimulating effect of pegbovigrastim administered to dairy cows on the leukocyte‐dependent immune response.^5^, ^8^, ^9^, ^11^, ^12^ A previous study^11^ demonstrated a more than 4‐fold increase in the number of circulating neutrophils 24 hours after the first administration of pegbovigrastim to cows before expected calving. The second injection administered after calving caused an approximately 10‐fold increase in the neutrophil count 1 day after injection when compared with the count in control cows.^11^ A marked stimulation of the production of total whole blood leukocytes and the neutrophil, monocyte, and basophil subsets was observed in another study after 2 administrations of pegbovigrastim to dairy cows during the transition period, which was especially evident after the second injection.^12^ Our observations are consistent with the results of the 2 studies, with a clearly stronger stimulation of leukocyte production manifested in the total and differential leukocyte counts, including that of granulocytes, after the second injection of pegbovigrastim compared with the baseline values in those studies.^11^, ^12^

Our previous study on the effect of pegbovigrastim injection on some immune parameters in calves challenged with *Mycoplasma bovis* showed similar changes in the number of circulating granulocytes and monocytes up to day 7 after first administration. This similarity occurred despite the blood collection time differing by 1 day at some time points (ie, being on day 4 instead of day 5, and day 11 instead of day 12).^14^ The effect of pegbovigrastim injection alone on the examined parameters was not further evaluated in the previous study because the calves were infected with a field strain of *M. bovis* on day 7, just before the re‐administration of pegbovigrastim.^14^ In our current study, after the second injection of pegbovigrastim, an increase in the numbers of not only granulocytes and monocytes but also of total leukocytes and lymphocytes (not determined in the previous study) was observed again and it was even greater than the increase induced by the first injection. The increase in lymphocyte count although progenitor cells were present which were different from granulocytes and mononuclear phagocytes could be due to the effect of pegbovigrastim on pluripotent hemopoietic stem cells in the bone marrow, which are the same for all leukocytes, or to an indirect low‐level stimulating effect of pro‐inflammatory cytokines on leukocyte production and release.^19^ Similarly, in other research, a significant increase in the number of circulating lymphocytes after both injections of pegbovigrastim also was observed in dairy cows during the transition period, although this increase was slightly delayed after the second administration.^12^ Some stimulating properties of pegbovigrastim administration in calves on leukocyte production and a concomitant circulating leukocyte count increase were confirmed in a previous study.^15^ Pegbovigrastim administered once to newborn calves at a dosage of 25 μg/kg bw effectively increased the number of the most commonly investigated leukocyte parameters (ie, total leukocytes, segmented neutrophils, band cells, and monocytes), but it did so with no significant changes in the lymphocyte and eosinophil counts.^15^ Despite some similarities, these results cannot be considered fully comparable with those of our current study because experimental design differed in the dose of pegbovigrastim, the number of doses, and the age of the animals used in the study.

In our current study, the increased phagocytosis of granulocytes and monocytes after administration of pegbovigrastim was evident in the significantly enhanced ingestion capability of individual phagocytes and the markedly prolonged phagocytic activity for monocytes. For comparison, in the pegbovigrastim‐treated calves previously studied, the individual phagocytic activity of granulocytes was less markedly augmented and that of monocytes was not affected.^14^ In contrast, in that study, pegbovigrastim administration significantly increased the percentage of phagocytic granulocytes. However, similar to our current study, previous research also found no significant changes in the percentage of phagocytic monocytes in the pegbovigrastim‐treated calves.^14^ In our current study, no significant effect of pegbovigrastim administration was observed on the oxidative burst of leukocytes. These results were consistent with those of our previous study with regard to the percentage of cells showing the ability to produce reactive oxidants, which was not significantly changed in the pegbovigrastim‐treated calves. In contrast, the enzymatic activity of the cells was enhanced, which was not observed in our current study.^14^ Those results can only be compared with those noted after the first injection of pegbovigrastim because, as already stated, on day 7 of the study (before re‐administration of pegbovigrastim) the treated calves were infected with *M. bovis*. Differences in the age of calves assigned to the experiments and differences of some time points of sampling also should be considered when comparing the results. The lack of a significant effect of pegbovigrastim administration to cows in the peripartum period on the phagocytic function of blood‐derived neutrophils was demonstrated in a previous study,^9^ although it should be stated that the authors did observe marked impairment of the phagocytic activity of individual cells on day 7 post‐calving. However, similar to our current study, no significant changes in the respiratory burst function of the neutrophils were found in the pegbovigrastim‐treated cows.^9^

In addition to phagocytosis and oxidative burst, neutrophils fulfill their antimicrobial function by degranulation, during which various enzymes are released, including MPO.^19^ Our current study showed a more stable MPO concentration in the serum of pegbovigrastim‐treated calves than in the control calves throughout the study. This finding may have been the result of the protective effect of pegbovigrastim on the calf against neutrophil degranulation, which is suggested by the clearly increasing values of this parameter at some time points in the controls which received no pegbovigrastim. Pegbovigrastim has a stable and beneficial effect on homeostatic relationships in the calf. A visible decrease in the optical densities of basal and stimulated MPO in the blood neutrophils of cows treated with pegbovigrastim twice during the peripartum period was shown in a previous study.^9^ In that study, increased release of MPO from the blood neutrophils in the pegbovigrastim‐treated cows was demonstrated, which preceded a decrease in this parameter at the next time point however.^9^

CD11b is the α‐subunit of 1 of the 3 β2‐integrin surface receptors—CD11b/CD18 (forming membrane‐associated component‐1, Mac‐1, MO‐1, complement receptor 3, CR3) which is expressed on bovine neutrophils and involved in cell migration into the site of inflammation or infection.^20^, ^21^, ^22^ In our current study, the percentage of CD11b was significantly increased after pegbovigrastim administration, and stimulation of this response was clearly identified for both injections. The results are consistent with in vitro observations in a previous study^3^ in which the expression of selected neutrophil adhesion molecules after cell incubation with bG‐CSF expressed in *Escherichia coli* was evaluated. In that study, a more than 2‐fold increase was observed in the geometric mean fluorescence values of CD11b after the treatment of isolated bovine neutrophils with recombinant bG‐CSF. Additionally, the expression of CD11a and CD11c, α‐subunits of the other 2 β2‐integrins, also was upregulated in the activated neutrophils. According to the authors, because of only 30 minutes of neutrophil incubation with rbG‐CSF, the upregulation of CD11b expression was probably the result of the recruitment of the stored integrin within the cell granules to the surface.^3^

Besides neutrophils, proinflammatory cytokines such as IL‐1β, IL‐6, and TNF‐α are involved in nonspecific immunity. These cytokines are necessary in small amounts to activate adaptive immunity, which makes the appropriate immune response to a specific antigen, and the mobilization of which follows the nonspecific immune response. These cytokines are also responsible for stimulation of many important functions supporting the host's defense against infection (ie, production, adhesion and migration of leukocytes).^19^ In our current study, a marked but not statistically significant increase in IL‐1β concentration was observed after the first pegbovigrastim administration. Similarly, the IL‐6 concentration was detectably increased after pegbovigrastim was first given and remained so after its second injection. Some increase in concentration of IL‐10 was observed only at the end of the study, which may indicate activation of anti‐inflammatory processes at that time.^19^ Our current results are in partial agreement with previous observations in a study^23^ that examined the effect of G‐CSF administration to human volunteers on the release of some pro‐ and anti‐inflammatory cytokines and cytokine receptors in stimulated blood. An increase in IL‐1RA release in the challenged blood of G‐CSF‐treated individuals was observed in both ex vivo and in vitro conditions and also regardless of the stimulator used. However, there were no significant changes in the ex vivo release of IL‐1β, in free TNF‐α, or in total TNF in the lipopolysaccharide (LPS)‐stimulated blood from G‐CSF‐treated donors. In that study,^23^ an increase in the LPS‐inducible ex vivo release of IL‐6 and IL‐10 also was demonstrated. According to the authors, G‐CSF has an immunomodulating effect on the cytokine response profile, but with a shift toward those cytokines with antiinflammatory properties, such as IL‐1RA and IL‐10, which was partly confirmed in our current study.^23^

## 5 CONCLUSIONS

Pegbovigrastim administration to calves caused a detectable increase in total and differential leukocyte counts, stimulation of the phagocytic activity of granulocytes and monocytes, and modulation of the response of selected cytokines. Our results documented stimulation of nonspecific immunity in pegbovigrastim‐treated calves, which supports its use in the prevention of diseases in calves during early rearing. However, more clinical research is needed to determine whether an increased leukocyte count and potentiated function decrease clinical disease occurrence.

## CONFLICT OF INTEREST DECLARATION

Authors declare no conflict of interest.

## OFF‐LABEL ANTIMICROBIAL DECLARATION

Authors declare no off‐label use of antimicrobials.

## INSTITUTIONAL ANIMAL CARE AND USE COMMITTEE (IACUC) OR OTHER APPROVAL DECLARATION

Experimental procedures were carried out in accordance with the requirements of the Local Ethics Committee on Animal Experimentation of the University of Life Sciences in Lublin, Poland (Decision No. 33/2018 admitted 12 February 2018) which also meet the European Union standards.

## HUMAN ETHICS APPROVAL DECLARATION

Authors declare human ethics approval was not needed for this study.

## References

[1] Semerad CL , Liu F , Gregory AD , Stumpf K , Link DC . G‐CSF is an essential regulator of neutrophil trafficking from the bone marrow to the blood. Immunity. 2002;17:413‐423. 12387736 10.1016/s1074-7613(02)00424-7

[2] Roilides E , Walsh TJ , Pizzo PA , Rubin M . Granulocyte colony‐stimulating factor enhances the phagocytic and bactericidal activity of normal and defective human neutrophils. J Infect Dis. 1991;163:579‐583. 1704903 10.1093/infdis/163.3.579

[3] Heidari M , Harp JA , Kehrli ME Jr . Expression, purification, and in vitro biological activities of recombinant bovine granulocyte‐colony stimulating factor. Vet Immunol Immunopathol. 2001;81:45‐57. 11498246 10.1016/s0165-2427(01)00321-x

[4] Barca J , Meikle A , Bouman M , et al. Effect of pegbovigrastim on clinical mastitis and uterine disease during a full lactation in grazing dairy cows. PLoS One. 2021;16:e0252418. 34043727 10.1371/journal.pone.0252418 PMC8158865

[5] Canning P , Hassfurther R , TerHune T , Rogers K , Abbott S , Kolb D . Efficacy and clinical safety of pegbovigrastim for preventing naturally occurring clinical mastitis in periparturient primiparous and multiparous cows on US commercial dairies. J Dairy Sci. 2017;100:6504‐6515. 28601453 10.3168/jds.2017-12583

[6] Denis‐Robichaud J , Christophe M , Roy JP , et al. Randomized controlled trial of pegbovigrastim as an adjunct therapy for naturally occurring severe clinical mastitis cases in dairy cows. JDS Commun. 2021;2:398‐402. 36337107 10.3168/jdsc.2021-0137 PMC9623706

[7] Ruiz R , Tedeschi LO , Sepúlveda A . Investigation of the effect of pegbovigrastim on some periparturient immune disorders and performance in Mexican dairy herds. J Dairy Sci. 2017;100:3305‐3317. 28161183 10.3168/jds.2016-12003

[8] Barca J , Schukken YH , Meikle A . Increase in white blood cell counts by pegbovigrastim in primiparous and multiparous grazing dairy cows and the interaction with prepartum body condition score and non‐esterified fatty acids concentration. PLoS One. 2021;16:e0245149. 33411851 10.1371/journal.pone.0245149 PMC7790418

[9] McDougall S , LeBlanc SJ , Heiser A . Effect of prepartum energy balance on neutrophil function following pegbovigrastim treatment in periparturient cows. J Dairy Sci. 2017;100:7478‐7492. 28647326 10.3168/jds.2017-12786

[10] Putz EJ , Eder JM , Reinhardt TA , Sacco RE , Casas E , Lippolis JD . Differential phenotype of immune cells in blood and milk following pegylated granulocyte colony‐stimulating factor therapy during a chronic *Staphylococcus aureus* infection in lactating Holsteins. J Dairy Sci. 2019;102:9268‐9284. 31400902 10.3168/jds.2019-16448

[11] van Schyndel SJ , Carrier J , Bogado Pascottini O , et al. The effect of pegbovigrastim on circulating neutrophil count in dairy cattle: a randomized controlled trial. PloS One. 2018;13:e0198701. 29953439 10.1371/journal.pone.0198701 PMC6023130

[12] Trimboli F , Morittu VM , Di Loria A , et al. Effect of pegbovigrastim on hematological profile of Simmental dairy cows during the transition period. Animals. 2019;9:841. 31640199 10.3390/ani9100841 PMC6826567

[13] Dudek K , Bednarek D , Ayling RD , Kycko A , Reichert M . Preliminary study on the effects of enrofloxacin, flunixin meglumine and pegbovigrastim on *Mycoplasma bovis* pneumonia. BMC Vet Res. 2019;15:371. 31655595 10.1186/s12917-019-2122-3 PMC6815429

[14] Dudek K , Szacawa E , Wasiak M , Bednarek D , Reichert M . The effect of pegbovigrastim injection on phagocytic and oxidative burst activities of peripheral blood granulocytes and monocytes in calves challenged with *Mycoplasma bovis* . Pathogens. 2022;11:1317. 36365068 10.3390/pathogens11111317 PMC9693237

[15] Kegles F , Madruga OC , Schmoeller E , et al. Hematological and biochemical parameters of dairy calves submitted to pegbovigrastim administration. J Dairy Sci. 2019;102:547‐556. 30527989 10.3168/jds.2018-14445

[16] Dudek K , Bednarek D , Szacawa E , Rosales RS , Ayling RD . Flow cytometry follow‐up analysis of peripheral blood leukocyte subpopulations in calves experimentally infected with field isolates of *Mycoplasma bovis* . Acta Vet Hung. 2015;63:167‐178. 26051255 10.1556/AVet.2015.014

[17] Wojcicka‐Lorenowicz K , Kostro K , Lisiecka U , Gąsiorek B . Phagocytic activity and oxygen metabolism of peripheral blood granulocytes from rabbits experimentally infected with *Trichophyton mentagrophytes* . J Vet Res. 2018;62:43‐48. 29978126 10.1515/jvetres-2018-0006 PMC5957460

[18] Dudek K , Bednarek D , Lisiecka U , et al. Analysis of the leukocyte response in calves suffered from *Mycoplasma bovis* pneumonia. Pathogens. 2020;9:407. 32456293 10.3390/pathogens9050407 PMC7281192

[19] Chase CCL . The essentials: the who, what, and where of the bovine immune system. In: Chase CCL , Walsh C , Casademunt S , et al., eds. Bovine Immunity: Making Immunology and Vaccinology Come Alive. 1st ed. Spain: HIPRA, S. A; 2022:2‐29.

[20] Cox E , Mast J , MacHugh N , Schwenger B , Goddeeris BM . Expression of beta 2 integrins on blood leukocytes of cows with or without bovine leukocyte adhesion deficiency. Vet Immunol Immunopathol. 1997;58:249‐263. 9436269 10.1016/s0165-2427(97)00027-5

[21] Smits E , Burvenich C , Guidry AJ , Massart‐Leën A . Adhesion receptor CD11b/CD18 contributes to neutrophil diapedesis across the bovine blood‐milk barrier. Vet Immunol Immunopathol. 2000;73:255‐265. 10713339 10.1016/s0165-2427(00)00157-4

[22] Diez‐Fraile A , Meyer E , Burvenich C . Regulation of adhesion molecules on circulating neutrophils during coliform mastitis and their possible immunomodulation with drugs. Vet Immunol Immunopathol. 2002;86:1‐10. 11943325 10.1016/s0165-2427(01)00432-9

[23] Hartung T , Döcke WD , Gantner F , et al. Effect of granulocyte colony‐stimulating factor treatment on ex vivo blood cytokine response in human volunteers. Blood. 1995;85:2482‐2489. 7537116

